# Engineering a sustainable future for point-of-care diagnostics and single-use microfluidic devices

**DOI:** 10.1039/d2lc00380e

**Published:** 2022-06-23

**Authors:** Alfredo Edoardo Ongaro, Zibusiso Ndlovu, Elodie Sollier, Collins Otieno, Pascale Ondoa, Alice Street, Maïwenn Kersaudy-Kerhoas

**Affiliations:** The Institute of Photonic Sciences (ICFO) Barcelona Spain; Medecins Sans Frontières (MSF), Southern Africa Medical Unit (SAMU) Cape Town South Africa; Stratec Consumables Anif Austria; African Society for Laboratory Medicine (ASLM) Addis Ababa Ethiopia; School of Social and Political Sciences, University of Edinburgh Edinburgh UK; School of Engineering and Physical Sciences, Heriot-Watt University Edinburgh UK m.kersaudy-kerhoas@hw.ac.uk; Infection Medicine, College of Medicine and Veterinary Medicine University of Edinburgh Edinburgh UK

## Abstract

Single-use, disposable, point-of-care diagnostic devices carry great promise for global health, including meeting urgent needs for testing and diagnosis in places with limited laboratory facilities. Unfortunately, the production and disposal of single-use devices, whether in lateral flow assay, cartridges, cassettes, or lab-on-chip microfluidic format, also poses significant challenges for environmental and human health. Point-of-care devices are commonly manufactured from unsustainable polymeric materials derived from fossil sources. Their disposal often necessitates incineration to reduce infection risk, thereby creating additional release of CO_2_. Many devices also contain toxic chemicals, such as cyanide derivatives, that are damaging to environmental and human health if not disposed of safely. Yet, in the absence of government regulatory frameworks, safe and sustainable waste management for these novel medical devices is often left unaddressed. There is an urgent need to find novel solutions to avert environmental and human harm from these devices, especially in low- and middle-income countries where waste management infrastructure is often weak and where the use of point-of-care tests is projected to rise in coming years. We review here common materials used in the manufacture of single-use point-of-care diagnostic tests, examine the risks they pose to environmental and human health, and investigate replacement materials that can potentially reduce the impact of microfluidic devices on the production of harmful waste. We propose solutions available to point-of-care test developers to start embedding sustainability at an early stage in their design, and to reduce their non-renewable plastic consumption in research and product development.

## Abstract 2

Single-use, disposable, point-of-care diagnostic devices carry great promise for global health, but present some challenges in terms of environmental sustainability. This perspective covers solutions to embed sustainability at the R&D and product development stages.

## Graphical abstract

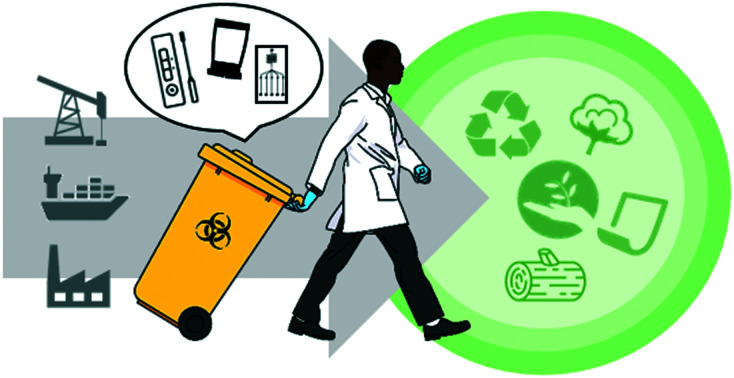

## Introduction

**Fig. 1:**
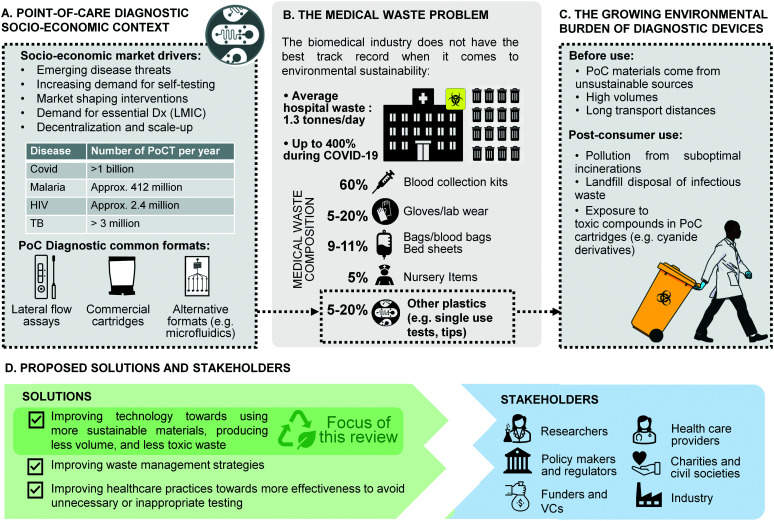
Overview of the challenges and solutions in single-use diagnostic devices. A) Point-of-care diagnostic socio-economic context.^13–15^ B) The medical waste problem.^16,17^ C) Growing burden of waste from diagnostic devices. D) Proposed solutions and stakeholders.

Nowhere has the increased availability of diagnostic tests for medical practice and public health been more evident than in the global response to the recent coronavirus (COVID-19) pandemic.^1,2^ Millions of point-of-care tests (POCTs) for COVID-19 are used globally every day in hospitals, primary care facilities, workplaces, and people's homes, bringing projections for the global POCT market to $72B by 2024 from $43.3B in 2022, at an annual growth rate of 10%.^3^ Heightened awareness of the benefits of POCTs following the COVID-19 response is also widely viewed as an opportunity to galvanise diagnostic innovation for neglected diseases and improve access to a wider array of tests in resource-limited settings (Fig. 1A).^4^

Yet the mass deployment of POCT devices in health systems and communities across the globe comes with unforeseen costs for the environment and human health. Most single-use POCT devices are made from plastic materials issued from non-renewable sources, and contribute to the rising global tide of medical waste (Fig. 1B). A large proportion of POCT diagnostic waste falls into the category of infectious waste, which should be collected separately and treated in order to remove the infection risk.^5^ Infectious waste is most often incinerated, thus contributing to greenhouse gas (GHG) emissions. In low- and middle-income countries (LMICs), many health facilities either lack incinerators altogether, do not have the fuel to run them, or cannot operate them at required temperature thresholds.^6–8^ In such settings, used testing devices are often burned on open pits in health facility grounds or at municipal dump sites.^5,9^ In addition to causing the release of GHG, plastic waste burned at low temperatures emits toxic pollutants such as dioxins and furans.^10^ In addition, a lot of POC waste in LMICs ends up in landfills or in municipal water supplies, which increases the risk that health workers, waste workers and members of the public will come into contact with the hazardous reagents they contain, such as the cyanide derivatives used in PCR cartridges (Fig. 1C).^11^ A recent WHO report estimated that during the COVID-19 pandemic more than 140 million test kits have been shipped through the UN procurement portal alone, with the potential to generate 731 000 litres of chemical waste, the equivalent of a 25 m 8-lane swimming pool.^12^ So while single-use POCTs undoubtedly carry great potential for global health, they also contribute to growing global challenges related to plastic waste, GHG emissions, and human exposure to toxic pollutants (Fig. 1C). Concerns about contaminated medical waste resulting in infectious disease spill over into animal populations (reverse zoonoses) have also been reported previously, including recently in the 2022 monkey pox epidemic.^18,19^ Moreover, in LMICs, increased access to POCTs in the future will place significant additional pressures on already stretched waste management systems, undermining claims that such devices are ‘infrastructure-light’ and appropriately designed for such settings.^20^ To a lower but growing extent, such pressure is seen in high-income countries as well, where environmental impact and waste management have become a recurrent topic at funding levels and where limited access to plastic during the pandemics triggered strategic discussions on consumable recycling. This is exemplified for example, by the wording of the European Green Deal and a number of European Commission funds calling specifically for CO_2_ reduction, and reduced water and energy usages across the full product life cycle.^21^

Meanwhile, advances in microfluidics, the technology of microscale fluid manipulation, are rapidly expanding the capabilities and reach of medical testing. A general trend in healthcare towards personalised, remote POCT procedures, and global concerns about emerging diseases are driving substantial investments in microfluidic innovation and rapid market growth in the diagnostics sector.^22–26^ As a consequence, point-of-care and microfluidic testing devices are now an essential component of disease control programmes, at the national and global level, from efforts to improve universal health care, to disease elimination campaigns and outbreak response.^27^

Until recently there has been little incentive for all actors (researchers, engineers, manufacturers) to develop and use more sustainable and less harmful materials in POCTs and single-use microfluidic devices. The Covid-19 pandemic had put a beneficial spotlight on medical waste issues, and solutions minimising PPE waste, as well as seminal frugal diagnostic solutions, have emerged recently.^28–30^ However, the considerations for safe and sustainable disposal have typically been excluded from design requirements so far. Accuracy, reliability, usability, and affordability are the main drivers in the industrial medical device sector. For example, the standard format for target product profiles (TPPs), which provide guidance to manufacturers on market needs and appropriate technical specifications, omits specifications for waste management. Global health policy efforts to address the rising challenge of healthcare waste in LMICs have tended to focus on improving country-level waste management regulation, monitoring and infrastructure rather than considering how the volume of healthcare waste might be reduced through improvements in design and manufacture earlier in the product life cycle. But improving waste management at the point of use can only take us so far, especially since the circular solutions in the medical diagnostic area will always be limited by the requirements for safe disposal of infectious waste.

However, change is on the horizon. Regulators around the world are now requiring more sustainability for single-use products, as laid out by public procurement approaches such as the United States' BioPreferred Programme^31^ and the EU's Green Public Procurement (GPP) framework.^32^ The Horizon EU grant scheme urges applicants to consider such life cycle, recycling and environmental impact. In the US, the Environmental Protection Agency (EPA) now requires that healthcare facilities under the umbrella of the federal government give preference to sustainable products. International financial institutions such as the Asian Development Bank and global health funding mechanisms such as the Global Fund make similar requirements for recipients to consider green procurement.^33^ But this pressure on health procurement agencies will not bear any positive outcomes, if there are no alternative sustainable products available. Furthermore, the focus on sustainability has not been accompanied by an equivalent regulatory pressure to reduce the risk of harm from toxic reagents in places where safe disposal is not possible. In this context, the design of POCT technologies for safe and sustainable disposal is both an ethical imperative for industry, and an opportunity for scientific innovation (Fig. 1D).

In this review, we use the term ‘POCT’ as an umbrella term to describe a range of single-use, portable tests and microfluidic-based or miniaturised devices. Devices like lateral flow assays and other devices meant to be used outside typical care settings should, strictly speaking, be referred to as ‘point of need’, rather than ‘point of care’. In addition ‘single-use’ devices can also be applied to the growing proliferation of purposes for such devices including food testing^34^ and environmental monitoring.^35^ In engineering contexts, microfluidic devices are also referred to as ‘micro-total analytical systems’, or ‘lab-on-a-chip’, and there are often blurry definitional boundaries between these different terms.

This review brings together perspectives from public health, social science, material science, microfluidic engineering and manufacturing to address the sustainability challenges posed by POCTs and explore technological solutions towards the improvement of POCT and single-use microfluidic sustainability (Fig. 1D). First, we give an overview of plastic materials currently used in the fabrication and manufacture of POCTs, and introduce more sustainable and less harmful alternatives to these. Then, we focus on the environmental and human health risks associated with many reagents used in POCTs, and provide a roadmap for all stakeholders in the sector.

## Current plastics used in single-use diagnostic devices and sustainable alternatives

The requirements for materials in single-use POCT devices include, besides obviously performance and reproducibility: (i) compatibility with mass-manufacturing processes; (ii) chemical and mechanical resistance; (iii) impermeability; and (iv) low cost. These requirements have driven the use of glass and plastic in the prototyping and manufacturing of devices at R&D and the commercial level. An industry survey based on a sample of selected microfluidic companies revealed that 59% of all commercially available devices are made of plastics (mainly thermoplastics), 12% are of glass, 12% of papers, 6% of elastomers and 6% of epoxy resins.^36^ In academia, our own survey showed that 55% of published devices are made of polydimethylsiloxane (PDMS), 12% of silicon and glass, 20% of thermoplastic materials, and 13% of paper. Current plastics involved in the fabrication of POC devices are listed in Table 1, alongside their applications, pros and cons. The thermoplastics used in both settings include, but are not limited to: polymethylmethacrylate (PMMA), cyclic olefin copolymer (COC), cyclic olefin polymer (COP), polypropylene (PP), acrylonitrile butadiene styrene (ABS), and high impact polystyrene (HIPS). These materials answer the main requirements for single-use devices, but have a large CO_2_ footprint and are non-biodegradable.

**Table 1:** Conventional materials for the fabrication of single-use POCT, LOC and microfluidic devices

Families of material	Associated prototyping and fabrication method	Advantages	Disadvantages	Ref.
Silicon and glass	Standard photolithography and soft lithography	• Thermal conductivity	• Higher cost of fabrication	Foret, 2013;^36^ Wlodarczyk, 2019^37^
		• Stable electro-osmotic mobility	• Dangerous chemicals involved	
		• Resistance to organic solvent		
Thermoplastics (*e.g.* PMMA, PC, PS, PET, PVC, ABS, COC, COP)	Injection moulding; fusion deposition modelling; laser cutting	• Resistance to alcohols	• Unsustainable source of raw materials	Becker, 2002;^16^ Morgan, 2016;^38^ Liga, 2016;^39^ Attia, 2009^40^
		• Mostly low cost	• Toxic fumes when incomplete combustion	
		• Rapid prototyping		
		• Mechanical recycling		
Elastomers	Casting roll-to-roll	• Easy and low cost of microfabrication	• Incompatibility with organic solvents	Friend, 2010;^41^ Hiltunen, 2018^42^
		• High elasticity	• Absorption of hydrophobic and small molecules	
		• Gas permeable		
Hybrids	Combination of the above methods	• Integration of functionalities	• High cost of fabrication	Sanjay, 2016^43^

**Fig. 2:**
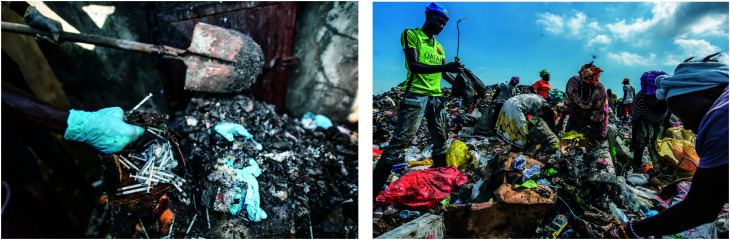
Left: Operator moving waste after incineration in a public referral hospital, Sierra Leone. Right: People at public rubbish dump pick through the medical waste from a Community Health Post (CHC), Western Rural Area, Sierra Leone. ©Olivia Acland/DiaDev.

Further, when improperly incinerated, these plastics can generate toxic pollutants. Fig. 2 illustrates sub-optimal incineration observed in a public referral hospital in Sierra Leone. Incineration is a high-temperature, dry oxidation process that reduces organic and combustible waste to inorganic, incombustible matter and results in a significant reduction of waste volume and weight. Incineration is an environmentally damaging process that releases combustion by-products into the atmosphere and generates residual ash. Such by-products include nitrous oxide as well as known carcinogens, which include polychlorinated biphenyls, furans and dioxins.^44^ Persistent organic pollutants such as polychlorinated dioxins and furans from halogenated plastics (such as polyvinyl chloride, PVC) are toxic at extremely low concentrations. While highly sophisticated incineration systems fitted with filters are capable of removing dioxins and furans, rudimentary unfiltered, homemade systems, which are used in some low-resource settings, will not. In addition, when dumped in landfills (Fig. 2), plastics will persist for hundreds of years in the soil. The sustainability and safety of single-use POCTs could, to various degrees, be improved by technological solutions.

### Recycled plastics

Recycling is a process that converts waste materials into new materials (primary to tertiary recycling) or energy (quaternary recycling).^45^ The adoption of recycled material for the production of POCTs could result in lower GHG emissions and reduce reliance on non-renewables such as petroleum. In particular, the most commonly used thermoplastics can be recycled by adopting one of three approaches: direct re-use, mechanical recycling or chemical recycling.

- *Direct re-use* involves re-using devices following a wash. This solution is unsuitable for most POCT, due to the presence of intricate features such as nano or microchannels, valves and other complex geometries, which can easily trap debris or get clogged during the wash-out, leading to assay failure, cross-contamination, and erroneous results.

- *Mechanical recycling* involves the collection, sorting, washing and grinding of the material, followed by mechanical processing of the waste into a secondary product, for instance *via* melting, remoulding and extrusion.^46^ This approach is typically conducted during either post-industrial processing (where it is most effective) or the post-customer use stage. In this case, scraps of the materials are immediately collected after the polymer processing, for example from the sprue and runners (passage through the liquid material is introduced into the mould and from one part to another) at each cycle of injection moulding. These can be reprocessed as they can be blended with the virgin material, to produce fallout products or other functional parts. The recycled end-item does not require a sorting or cleaning process and its chemical composition and properties are known. On the other hand, post-customer use mechanical recycling is more challenging and has some limitations. Collection, sorting and washing are fundamental steps. In fact, polymer blends (mixtures of two or more different polymers) usually have reduced mechanical and thermal properties when compared with those of the single polymer. To overcome this issue, additives in the form of compatibilizing agents are often added.^47–49^ Some research studies have shown the possibility of using mechanically recycled PMMA for the production of optical fiber sensors and microfluidic devices.^50,51^ The waste generated and the demand for new raw materials are decreased, reducing energy usage, air pollution and water pollution. Wan *et al.* designed a novel method to recycle PMMA microfluidic devices in a laboratory setting, demonstrating that recycled plastics can be used for the production of microfluidic devices. They showed the possibility to thermo-mechanically recycle PMMA up to 4 iterations without losing optical properties and biocompatibility (Fig. 2).^51^ Despite optimum results in terms of final optical qualities and thermal properties, these processes require specific facilities, such as an extruder or a heated press. In the case of single-use medical devices, a sterilization step should be included prior to mechanical recycling of the device. Another emerging material of interest, that can easily be mechanically recycled, is the thermoplastic elastomer Flexdyme™.^52,53^ Flexdym™ is the first material to be created specifically for the microfluidic community. It combines the advantages of thermoplastics and elastomers and is free of additives, which makes it ideal for sensitive cell culture applications for example. Flexdym TM can be molded through hot embossing or injection moulding and easily remolded.

- *Chemical recycling* is a closed-loop recycling process. First, the used thermoplastic is depolymerized through a chemical process such as chemolysis or pyrolysis, to break the macromolecular chains. After a distillation process, all impurities are separated to obtain the recycled monomer with a purity up to 99.8%. The monomers are polymerized to obtain the recycled material with the same optical, mechanical and thermal properties as the virgin material. Chemically recycled thermoplastics are widely accessible. For example, chemically recycled PMMA is available from several suppliers and constitutes a sustainable and flexible material (it can be cut, engraved, milled and embossed). Plasticizers and compatibilizing agents can change the material's thermo-mechanical properties and solvent affinity, which can affect prototyping protocols, such as laser microstructuring or bonding. This is an issue encountered with recycled as well as non-recycled virgin materials. We demonstrated an ultra-fast bonding method on pristine PMMA sheets manufactured from different suppliers and showed significant bonding strength differences between manufacturers.^46,54^ We have also shown that chemically recycled PMMA yielded similar bonding strength to the pristine PMMA material.^55^ Carrying out a Differential Scanning Calorimetry (DSC) analysis can help to predict the presence of contaminants and establish if further optimization of the bonding parameters—in terms of temperature, pressure and time—is required.^55^

Finally, it is worth mentioning here that another thermoplastic polymer, which is widely employed, and for which there is already a recycling stream route, is polyethylene terephthalate (PET). PET is the same polymer used in the plastic bottle industry. Unlike PMMA, PET is not commonly used in the production of microfluidic devices, but a few examples have been demonstrated: Jackson *et al.* used PET to fabricate semi-automated DNA extraction on a centrifugal device using mixing *via* an external magnetic field.^56,57^ The reason why PET has still not been widely adopted by microfluidic researchers and manufacturers might be due to the fact that it is not easily available in sheet format. Fabrication technologies of microfluidic devices from hard plastic employed both in academia and industry rely on CNC milling and laser cutting, which require a sheet format substrate. Still, recycled PET is widely available and could be seriously considered as a substitute to thermoplastics.

Recycled plastics might offer the possibility of approaching net zero CO_2_ emissions. While commercially recycled plastics are more expensive at present (re-PMMA is about 20% more expensive than pristine PMMA, for instance), this could be offset by higher costs on pristine plastic products in the future, and further incentives on the use of recycled components in single-use device production. More research into the use of recycled plastics in microfluidic production is needed and manufacturers could help by supplying in-depth material information to accelerate the optimisation of manufacturing processes, such as moulding, engraving and bonding.

While reducing GHG emissions, recycled plastics do not entirely remove the reliance on non-renewable raw material sources, nor do they alleviate the problem of pollutants generated by incineration. The use of recycled plastics, such as re-PMMA, represents a suitable short-term solution for some of the environmental challenges highlighted but the need for specific infrastructure, the logistics required to collect and recover waste, and the cost involved in recycling PMMA (as opposed to other thermoplastic materials such as HDPE and PET, for which a recycling stream already exists) make this option challenging in many settings. Regrettably, recycling is not always an economically advantageous option, and up to 30–50% of plastic waste cannot be recycled. Recycled plastics' pros and cons are summarised in Table 2, alongside other sustainable alternatives to plastic non-renewable plastic sources.

**Table 2:** Emerging and sustainable materials for the fabrication of single-use POCT, LOC and microfluidic devices

Families of material	Specific materials	Associated prototyping and fabrication method	Demonstrators	Advantages	Disadvantages	Ref.
Recycled plastics	Re-PMMA	Laser cutting, embossing, injection moulding	Simple microchannel structures; cell culture	• Readily available	• Non-renewable raw material	Ongaro, 2018;^55^ Wan, 2017^51^
				• Compatible with conventional manufacturing	• Poor degradability	
				• Good transparency	• Use of plasticizers aggravates incineration pollution	
				• Low autofluorescence		
				• Easily recyclable		
Bio-derived and biodegradable plastics	Shellac	Hot-embossing	Simple microchannel structures	• Bio-derived and biodegradable	• No transparency	Lausecker, 2016^58^
	Zein	Hot-embossing	Microfluidic gradients	• Bio-derived and biodegradable	• No transparency	Hsiao 2011^59^
	PLA	3D printing, laser cutting, injection moulding	Droplet, mixers, DNA melting, cell culture, protein analysis	• Good transparency	• No sheets available commercially	Tsuda, 2015;^60^ Tothill, 2017;^61^ Ongaro, 2018;^62^ Romanov, 2018;^63^ Ongaro, 2020^64^
				• Low autofluorescence		
				• Mechanical recycling		
Natural fibrous materials	Paper	Wax printing	Lateral flow immunoassays; DNA-based assays; blood typing	• Low cost	• 2D microfluidic device	Martinez, 2010;^65^ Carrell, 2019;^66^ Reboud 2019^67^
				• Light weight	• No transparency	
				•Readily available	• Limited volume capacity	
				•Easily recyclable		
	Wood	Laser cutting	Simple microfluidic structures; protein assay	• Low cost	•Material with inherent biological, chemical and mechanical variability	Andar 2019;^68^ Brigham 2018^69^
				• More rigid than paper		
	Cotton	Coating; laser writing	Immunoassay, colorimetry, wearable, blood microsampling	• Low cost	• Fragility	Wu, 2015;^89^ Ulum 2016;^71^ Xiao 2019;^72^ Stojanović, 2020^73^
				• High flexibility	• No transparency	
				• Amenable to wearable applications		

### Bio-based polymers

Bio-based plastics are polymers derived from organic biomass sources and may represent an alternative to recycled plastics.^74–76^ Biodegradable plastics are in line with the European Plastics Strategy, which aims to substitute polymers from fossil fuel-based resources with more environmentally friendly alternatives. The choice of bio-derived biodegradable material can strongly decrease the potential environmental footprint associated with raw material extraction, as well as reducing some of the environmental and human health concerns associated with incineration. In this section we review studies using bio-derived materials for microfluidic production.

#### Shellac

Wallrabe *et al.* chose shellac, a thermoplastic natural polymer secreted by the female lac bug *Kerria lacca*, to reduce the environmental impact of microfluidic devices, at the production, usage and end-of-life stage.^58^ Before the 1950s, shellac was widely used for the high-volume manufacturing of consumables—for example, to produce phonographs, before being replaced by PVC in 1948. Nowadays, shellac is used as a coating material for pills to enable slow drug release, as a food additive and as a dielectric layer to produce environmentally friendly transistors. Thanks to its low glass transition temperature (*T*_g_ of 42 °C), shellac could enable energy efficient hot embossing. However, due to its brittle nature, the shellac solution was deposited onto a paper substrate to achieve adequate mechanical properties for the hot embossing process and microfluidic structure imprinting on the shellac substrate. The authors successfully fabricated a POCT device using only materials from renewable resources using a low-energy method (around 18 kW h kg^−1^). For now, this process can only be used to prototype 2D structures and the integration of different components, such as electrodes or membranes, may be challenging. Also the use of paper to provide mechanical support can limit the number of suitable applications. For instance, fluorescence analysis is hindered because of the high auto-fluorescence of paper. However, shellac remains a renewable and sustainable material to consider in the future.

#### Zein

Hsiao *et al.* proposed zein, a polyamine protein found in maize, as a new sustainable substrate material for microfluidic applications,^59^ especially when a great number of samples and/or trials are required. Zein is composed of many amino acids, and currently used in the food industry to coat candies, nuts, fruits and pills as well as in food packaging and adhesives.^77^ Zein was selected for its excellent manufacturability in film shapes, with the possibility to tune and engineer the final substrate properties by changing the processing parameters. They successfully manufactured several complex microfluidic structures *via* solvent casting and proved good bonding strength to different substrates without any leakage. However, when in contact with water, protein precipitation decreased the substrate transparency, leading the authors to bond the microstructured zein substrate to glass. While transparency is not an issue for most devices placed in the market (especially for POCT applications), this is not true when optical detection is needed, either to check the final design (optical quality control) or for analytical purposes (for example cell sorting and imaging).

#### Polylactic acid

Polylactic acid or polylactide (PLA) is a thermoplastic produced from starch. As one of the most commonly available plant-based bioplastics, PLA can be recycled, both mechanically and chemically, or biodegraded under industrial composting conditions (with industrial infrastructure). Lifecycle assessment (LCA) calculations show that PLA reduces GHG emissions by up to 40% and reduces non-renewable energy use by up to 25% compared to fossil derived polymer alternatives.^78,79^ Research is under way to explore non-food crop renewable sources of carbon, such as lignocellulosic sources, algae, direct CO_2_ capture and waste (biomass) sources.

PLA, with its high biocompatibility and bioresorbability, has found applications in tissue engineering applications, food packaging and drug delivery. As a polyester alpha, it can be processed *via* injection moulding, extrusion, hot embossing, solvent casting and film blowing and has gained more and more attention after the development of fusion filament deposition 3D printing in desktop 3D printers. In addition to having medical grade properties, PLA is relatively cheap ($1.58 per kg as of January 2022), making this bio-based and biodegradable polymer attractive to the POCT market. A number of scientific providers have started proposing biodegradable consumables: for example, Sigma Aldrich sells plastic stirrers and spoons made of PLA.

PLA has been demonstrated in microfluidic applications using prototyping techniques such as 3D printing and laser cutting. A laser cutting and layer-by-layer lamination approach allows flexibility in the design, is user-friendly and low-cost, does not require the need for post-treatment and can be applied to an almost unlimited number of materials.^80,81^ This approach also enables surface and local treatments, and integration of various complex elements, such as membranes or electrodes.

We have pioneered techniques for the use of PLA in single-use microfluidic devices with well-controlled characteristics.^62,64^ Our work has shown the possibility to microstructure complex PLA-based microfluidic devices in a few minutes. The devices have shown better performance with respect to qPCR inhibition in comparison with PMMA; good transparency without the need to integrate optical windows; better biocompatibility than other typical thermoplastic materials employed for microfluidic cell culture or organ-on-a-chip;^64^ no absorption or adsorption of small molecules; and the possibility of integrating graphene water ink-printed electrodes to perform electrochemical analysis.^62^

Furthermore, advances in 3D printing technologies in the last five years have enabled fabrication of PLA-based devices on a scale compatible with microfluidic features. For example, Tothill *et al.* manufactured a 3D-printed device for a glucose assay.^61^ Despite very promising results, the fabrication of 3D-printed PLA microfluidic devices uses fused deposition modeling (FDM), which still has some limitations, such as printing time (resulting in low throughput), low resolution, poor surface finish and the need for post-treatment. Moreover, a good transparency has not yet been achieved, which can limit applications, thus necessitating imaging. Some research has been carried out in order to overcome this transparency issue.^63,82,83^

To conclude, PLA has been demonstrated as a new suitable substrate material for the production of environmentally sustainable microfluidic devices for POCT applications and shown to be compatible with prototyping techniques such as 3D printing and laser cutting. In addition, PLA can be injection moulded, and prototyped structures *via* FDM, or layer-by-layer assembly, should be easily adaptable to mass manufacture.

With incineration as the end-of-life scenario, the environmental benefit of biodegradable bioderived polymers lies in the use of renewable raw materials. In a landfill scenario, in addition to the raw materials, the benefits will also lie in fast degradation. However, unlike shellac or zein,^84,85^ PLA does not readily degrade in the natural environment.^86^ PLA biodegradation necessitates specific waste management, such as segregated collection and industrial composting facilities. While this is feasible and thus of interest for high-income countries, in an LMIC context, and considering landfills, PLA solutions would have no other positive environmental impact, beyond the use of renewable raw materials.

The transition from fossil-based to bio-based or recycled substrate materials represents, in the field of point-of-care and lab-on-chip, only a small step towards the goal of reaching truly sustainable solutions. A mere material substitution might not ease the environmental burden associated with the POCT, and water consumption and cradle-to-gate equivalent CO_2_ consumption per tonne of material produced should be taken into consideration when considering a material substitution. A recent review of bioplastics offers a comprehensive overview about the challenges and opportunities offered by these new materials.^87^ More research on the matter, outreach activities to raise awareness,^88^ clear regulations, and financial incentives will be pivotal to ensure the adoption of sustainable solutions that are adequate from both environmental and economical perspectives.

### Natural fibres

#### Paper

Cellulose is a water-insoluble homopolymer of glucose that is synthesized from plants or bacteria.^69^ Cellulose is one of the most abundant biomaterials on Earth, is low-cost and easy to manufacture, and is commonly used in clinical settings (*e.g.*, in dipstick tests). The concept of paper microfluidics, which makes use of cellulose as a substrate material for microfluidic devices, was introduced 10 years ago, developed with frugality at the very core of its rationale.^65^ Although the environmental sustainability arguments of paper microfluidics were not highlighted by those who originally developed the concept, cellulose is a highly renewable, lightweight material that is easily degradable and recyclable. Paper microfluidic devices, which can be used as lateral flow assays (LFAs), have been demonstrated to detect pathogens and biomarkers, utilising labelled antibodies to capture and detect biomolecules through visual, colorimetric or fluorometric readout, in numerous fields, from human diagnostics to environmental monitoring. In these assays, the fluid is transported *via* capillary action, without the requirement for fluid handling equipment.^66^ The fabrication methods of paper-based microfluidic devices involve wax patterning, inject printing, photolithography, plotting and laser treatment and are compatible with manufacturing at low-cost (typically ∼$0.50 per assay).^89^ Microwire electrodes can be incorporated into paper fluidic devices to provide highly sensitive and selective virus detection *via* electrochemical impedance spectroscopy.^90^ Paper devices are inherently limited in terms of fluid volumes and fluid phenomena, and are often deployed as lateral flow tests, which need operators to pipette reagents and samples in and out of the devices. However, paper microfluidic devices are increasingly deployed for complex point-of-care procedures, combining sample processing from whole blood together with an easy-to-read visualization for the rapid readout of diagnostic information. A recent example demonstrated precision diagnostics for malaria in low-resource, under-served settings, with a sensitivity higher than that of the malaria diagnostic tests currently in use.^63^ Paper microfluidics is compatible with simple prototyping techniques, which include hot wax printing and laser cutting, and should be easily translated to industrial techniques such as roll-to-roll manufacturing and die-cutting.

Of interest to this review, paper is also being used to replace the casings and non-cellulose pads in traditional LFAs. LIA Diagnostics was the first commercial company to propose an FDA-approved, flushable LFA pregnancy test, based on a 100% cellulose structure.^91^ More recently, Okos diagnostics has introduced a 100% cellulose-based LFA, although not commercialised nor FDA approved yet.^92^ Overall, we hope to see paper microfluidic devices implemented on mass manufacturing lines in the near future, replacing plastic-based and bulkier LFAs when appropriate.

#### Cotton

Cotton may form the substrate and functional material of next-generation POCTs. Cotton is a natural plant fiber which grows around the seed of the cotton plant, and is intensively farmed in the US, China and India for use in the textile industry. Li *et al.* firstly reported the use of cotton thread for biomarker detection in urine and plasma.^93^ Compared to paper, cotton threads naturally form microchannels and networks which can be easily built up with knots and entanglements, generating microfluidic circuits.^94^ The applications of cotton thread mainly lies in wearable biosensing devices so far, for the detection of glucose and for cytostatic drugs in sweat.^69,88^ The use of cotton has been limited by the presence of warp, weft threads, and holes; the fragility of the substrate makes it difficult to translate experimental manufacturing techniques to industrial mass manufacturing of biosensors or POC devices.^95^ However, recent advances in manufacturing on a single piece of cotton cloth have demonstrated the possibility to easily prototype devices with a hydrophilic channel resolution of 500 μm and a hydrophobic barrier of 100 μm for the detection of glucose and protein in urine in a high-throughput fashion. This is thanks to the combination of the low cost of cotton textiles and inexpensive high-throughput photolithography techniques.^70^ Cotton is a natural, renewable material similar to paper in its microfluidic qualities and low-cost: devices may start from $0.39.^86^ Thus, we envision that new inexpensive manufacturing technology will pave the way to new cotton-based microfluidic devices for POC where qualitative read-outs are needed, and for wearable applications.

#### Wood

Andar *et al.* have proposed a proof-of-concept microfluidic device made of birch plywood.^68^ In this work, channels were engraved in wood by means of a laser cutter and then coated in Teflon to counteract wicking. When used for protein detection by surface plasmon-coupled fluorescence enhancement, the device performed as well as or better than a plastic counterpart. These devices might be a step towards more environmentally friendly substrate materials in microfluidics. Natural vegetable oils and beeswax may be able to replace the Teflon coating in the future.^96^ Challenges in the control of the mechanical and chemical properties of wood products may nevertheless present challenges to the commercial applicability and use of such devices in advanced applications, such as biosensing. However, in a distant future, synthetic biology may be harnessed to grow natural materials like wood in a very controlled way, which would open the door for industrial applications.

### Reagents and sustainable alternatives

The problem of diagnostic waste goes well beyond the issue of how to replace or dispose of single-use plastics. Diagnostic tests often contain chemical reagents that need to be disposed of safely after use, dictated by local chemical waste disposal routes. Illegal or negligent disposal of chemical liquid waste in landfills and public sewage systems can lead to the contamination of groundwater and drinking water.^6,11^ Here we review some of the toxic waste associated with diagnostics, and possible alternatives.

#### Cell-fixing reagents

Biochemical testing often involves a fixation step in the sample preparation to meet biosafety requirements and prevent sample degradation. Fixation terminates any ongoing biochemical reaction to preserve sample structure, strength and stability. When moving to microfluidic automation, the most suitable fixation method relies on chemical fixation. Formaldehyde, belonging to the family of aldehydes, is the universal fixative solution in routine sample preparation, and the solution most commonly found in commercially available labelling kits.^97,98^ However, formaldehyde is a carcinogenic substance, can cause cytotoxic and genotoxic reactions and needs to be disposed of as chemical hazardous waste. Even though it can be used at very low concentrations, when incorporated in automated single-use microfluidic platforms, great care should be taken in terms of platform design to avoid spilling of reagents, and the single-use cartridge should be properly disposed of to avoid human and environmental risks.

Alcohols represent the second major class of fixation agents adopted. They represent a safer and cleaner alternative solution to aldehydes, with ethanol, methanol, denatured alcohol and isopropyl alcohol being appropriate replacements.^99^ Natural alternatives such as honey, sugar syrup and jaggery have all been successfully proposed.^100–103^ Sabarinath *et al.* reported that honey can be effectively used as a fixative when compared with formalin.^102^ They were able to image nuclear details both in honey and formalin without observing differences in subsequent staining protocols and microscopic morphology. However, the intrinsic viscosity of honey can make it a complicated material to work with at the microfluidic scale. Culinary sugar has been proposed by Patil *et al.* as a fixative agent, together with jaggery, and sugar syrup.^101^ They observed that jaggery fixation performed as well as standard formaldehyde fixation methods. Another study investigated substitution of formalin for commercially available and ‘home-made’ fixative solutions. Zanini *et al.* demonstrated that formaldehyde can be replaced by commercially available alternatives (RCL2 and CellBlock); custom fixatives including PAGA (polyethylene glycol, ethyl alcohol, glycerol, acetic acid); and two zinc-based fixatives (ZBF, Z7) as tested on more than 200 specimens.^104^ We envisage that more advances in the green chemistry field and future regulatory requirements for safer and cleaner products will help to generate new potential candidates to replace hazardous fixative reagents.

#### Nucleic acid extraction reagents

In the last 15 years, under the leadership of the WHO and some non-governmental organisations, the world has seen an unprecedented use of HIV viral load testing, which has contributed to considerable progress towards controlling the global HIV epidemic.^11^ However, a detrimental effect of this mass testing has been the generation of chemical waste. It has been estimated that about 924 000 litres of effluent chemical waste have been generated for HIV testing alone. Such tests, and many others, such as tuberculosis diagnostics, involve solid-phase nucleic acid extraction. Most kits use chaotropic agents made of cyanide derivatives, such as guanidinium thiocyanate.^105^ Cyanide salts are highly toxic both to human and aquatic life, and should not be flushed into drains or buried. These issues are compounded by the fact that contact between cyanide salts and oxidising agents like bleach can lead to the release of toxic cyanide gas.

Manufacturers recommend guanidinium thiocyanate to be disposed of according to country-specific rules, but countries where these POCTs are mostly needed often have a lack of waste regulations. Furthermore, safe disposal of cyanide derivatives involves incineration at a high temperature (850 °C) under specific conditions.^106^ Incineration facilities with such capabilities are not readily available, especially in lower-income countries. In one anecdotal instance, practitioners have reported driving to a local cement factory to safely dispose of the materials.^106^ Such arrangements are cumbersome, and unlikely to be followed by many institutions, which are operating under time, staffing and financial pressures. A number of calls have already been made to identify waste management solutions to these problems.^11^ However, better waste management can only address part of the problem and safer reagents are needed.

In order to avoid the use of chaotropic reagents, non-chaotropic methods have been developed as alternative techniques. Recently Merck introduced GenElute™-E Single Spin DNA and RNA Purification Kits specifically to address environmental issues. With this product they claim waste prevention (55% reduction in plastic), sustainable packaging and safer disposal (no hazardous liquid waste). The kit works on the principle of negative chromatography using size exclusion, which has been implemented on-chip before. Rodrigues *et al.* reported the use of sodium dodecyl sulphate (SDS) and acetyl trimethyl ammonium bromide as extraction reagents for the isolation of DNA from two types of fungal materials.^107^ In this work they also reported using vortexing with glass beads inside a closed tube for mechanical disruption of conidia cell walls. Aminosilane-modified surfaces,^108^ magnetic particles coated with APTES,^109^ or chitosan-coated beads^110^ have been proposed and demonstrated for DNA extraction on whole blood or *E. coli* cells. The dimethyl adipimidate (DMA)-based solid-phase extraction method resulted in successful extraction of DNA in whole blood and urine, but yielded a significantly lower DNA amount than the commercial benchmark using standard chaotropes.^111^

A number of safer and more environmentally friendly alternatives to chaotropic agents have been proven to be efficient for DNA extraction but remain behind standard chaotropic methods in terms of DNA yield.

### Roadmap to sustainable and safe POCT and LOC development

In order for the sustainability and safety challenges associated with the disposal of POCTs to be addressed, action is required across the product lifecycle. Significant investment is needed to strengthen waste management infrastructure in LMICs. More can also be done to strengthen regulatory requirements for green diagnostic products at a global and national level. However, researchers, product developers and manufacturers also need to play their part. Here, we discuss some guidelines to improve sustainability at multiple levels, from regulators to designers.

#### The role of regulators and policy makers

Often, the responsibility for diagnostic waste falls on national governments, as the majority of diagnostic test instructions for use (IFU) recommend disposal of waste according to local safety regulations. However, health waste management services, including regulatory frameworks and physical infrastructure, as well as expertise for efficient disposal of medical diagnostic waste, are lacking in many LMICs.^7^

Rather than place full responsibility for safe disposal of POCTs on users, there is a need for POC providers to contribute to solutions at the end of the product life-cycle. This includes improving the safety and sustainability of their products without decreasing the affordability of POCTs. To encourage such developments, TPPs published by the WHO and other international organizations, produced to guide technical specifications for future diagnostic tests, ought to provide dedicated guidance on acceptable features for waste disposal. Such guidance from TPPs can place emphasis on an expectation for the environmental footprint of such tests to be small, using either recyclable or compostable materials, without the need for high-temperature incineration. Advocacy work through civil society can also put pressure on POC providers to consider waste management at the design stage so as to minimize harm to human health and the environment.

POC test providers could also support initiatives to improve national waste management infrastructures and regulatory frameworks, including workforce training, in LMICs through either corporate social responsibility efforts or the adoption/enforcement of extended producer responsibility (EPR) mechanisms. The African Society for Laboratory Medicine (ASLM), through the Laboratory Systems Strengthening Community of Practice, could expand its regional influential role of monitoring national waste management systems in LMICs, coordinating and sharing resources to strengthen these whilst also linking POC providers, the WHO, *etc.*, with countries that need support.

Commendable efforts from Europe's primary regulator for materials, ECHA, on banning certain types of plastics from the market will go a long way towards curbing plastic waste^112^ and such efforts should be replicated by other regulatory agencies. In 2017, the WHO published the global model regulatory framework for medical devices, including *in vitro* diagnostic medical devices,^113^ and this framework emphasises that waste management practices should follow the manufacturer's instructions. The revised version of this guidance, which is still in production, must contain explicit language regarding the need for POCTs to have a small environmental footprint.

#### ‘Design for Sustainability’ approaches

When designing new devices, engineers can adopt design for sustainability (DfS) approaches.^114^ DfS is a design philosophy with the basic objective of achieving sustainability by considering a number of principles for sustainability at the design stage, rather than retrospectively—*i.e.* after production has taken place.^115^ One of the DfS methodologies can enable the integration of the ‘reduce–reuse–recycle’, or 3R principle.^115^ In the case of POCTs, their use should be employed only when necessary, thus avoiding overuse or favouring the ‘reduce’ approach. The reprocessing and ‘reusing’ of POCTs cannot be easily implemented due to cross-contamination issues. However, recycling might be possible in the future, as discussed. According to Clark *et al.*, DfS provides different methodologies to make sustainable improvements to products by adopting elements of lifecycle analysis, considering the whole lifecycle, from raw material extraction to final disposal.^116^

**Fig. 3:**
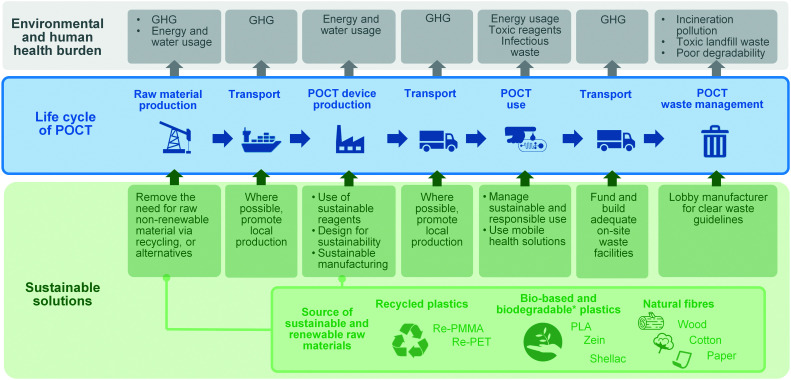
Overview of the life cycle of POCTs, POCT-associated environmental burden and sustainable solutions associated with each stage of the life-cycle.

The product life cycle starts with the extraction, processing and supply of the raw materials, to cover then the production of the product, its distribution, use (and possibly reuse and recycling), and its ultimate disposal. The life cycle of POCTs is illustrated in Fig. 3. Environmental impacts occur in different phases of the product lifecycle and should be accounted for in an integrated and holistic way. The DfS approach can be applied to medical microcomponents, taking into account the need to: (i) minimise non-renewable energy consumption; (ii) use environmentally preferable materials; (iii) protect and conserve water; (iv) minimise waste; and (v) lengthen the product shelf-life. In addition, in the context of sustainable POCT development, DfS principles should be expanded to minimise the potential for people to come into contact with harmful chemicals and take human risks associated with the disposal of POCTs into account.

A simple approach to DfS for POC engineers starts by thinking about beginning of life (*e.g.*, *where does the raw material come from?*) and end of life (*e.g.*, *what happens to the product once used?*). Designing diagnostic methods that use lower reagent volumes, or do more multiplexing to be able to run several tests in one reaction would be advantageous in terms of environmental sustainability and economic considerations, both of which are already embedded in microfluidic concepts. Engineers may consider if the product could be recycled and, if so, how can the design improve the chance of it being recycled. The Sustainability Guide project^117^ has produced a number of freely accessible DfS resources.

Researchers may be able to reduce the environmental impact of their approaches by considering their substrate materials and reagents. When possible, natural fibres, which promise to cut the plastic burden, should be used. A pragmatic approach for researchers and designers is to create a list of materials and reagents used in the fabrication of prototypes, their pros and cons, and consider whether sustainable alternatives are available or not. One straightforward substitution is pristine PMMA for recycled PMMA. If a research lab cannot afford the replacement, a good practice to encourage sustainability might be to flag the possibility in the publication materials and methods section. Researchers should consider recycling prototypes and ancillary plastics when possible. Complete recycling and re-use cycles for microfluidic chips have been demonstrated.^51^ If such re-use cycles are not possible, specialist services may be used to collect plastic waste and get it recycled externally. Only plastics that have not been in contact with infectious waste should be recycled, however, and researchers should make sure that robust health and safety protocols are in place for recycling activities.

As illustrated in Fig. 3, one of the main GHG emission sources in the life cycle of a POCT is transportation. Where possible, local materials should be considered over materials shipped from overseas. In addition to the contribution that global supply chains make to GHG emissions, the shortage of tests during the COVID-19 pandemic has shown the importance of resilience and local manufacturing at a basic research and industrial level. Thus when possible, local production and adequate on-site waste facilities should be promoted. Furthermore, local facilities might serve as POCT production sites at the point-of-need by implementing state-of-the-art rapid and agile technologies, such as 3D printing. Finally, mobile health, the application of mobile devices and related technologies to healthcare, is playing a significant role in under-served communities where infrastructure around transport and healthcare is lacking.^118–120^ Automatic smartphone-based diagnostic systems have been the subject of numerous investigations as well as several commercial endeavours.^118,121–123^ While mobile health solutions are sought for providing convenient point-of-care solutions, they also effectively reduce associated infrastructure, transport and logistics around diagnostics, thus mitigating the GHG emission of single use diagnostics. Where possible, mobile health solutions should be considered by developers as another way to improve the environmental sustainability of point-of-care diagnostics.

#### Life cycle analysis tools for industry

The next step after considering DfS approaches is to use a life cycle analysis (LCA). This is a formal, ISO-regulated method, used to evaluate the environmental impact of a product through its lifecycle, and encompasses the extraction and processing of raw materials, manufacturing, distribution, use, recycling and final disposal.^124^ LCA is generally carried out by specialists in academia or by production engineers in industry. LCA features four main components: (i) goal and scope, which involves setting boundaries for the study; (ii) the inventory, which includes gathering data including relating to emissions, energy requirements and material flows for each process involved; (iii) impact assessment, where impacts on the environment (usually water consumption, CO_2_ generation and ozone layer depletion) are calculated; and (iv) improvement assessment. Microfluidic engineers are encouraged to build collaborations with LCA specialists. OpenLCA, LCA calculator and SimaPro® are free online LCA tools to perform comprehensive LCA of products and manufacturing processes. Through its holistic, fact-based and formal approach, LCA can help progress product sustainability at the industrial level.

#### Circularity prospects for medical single-use plastics

A circular economy is defined as a model of production and consumption, which involves reusing or recycling existing materials and products as long as possible with the goal to reduce reliance on raw materials. Due to issues surrounding health and safety regulations, the circular economy model has seldom been applied in the health and care sector, apart from limited trials for PPE, or medical electronic equipment.^125–127^ When, as in the case of point-of-care diagnostics, single-use cannot be avoided, there is still the potential for recyclable materials to be sterilised, disassembled, segregated and collected for chemical recycling. A number of commercial systems already offer a waste-to-pellet solution to transform medical plastic waste into a sellable by-product (so far often limited to polypropylene). Such systems, for example Pharmafilter^128^ and EcoSteryl^129^ systems, include a sterilisation step and can be combined to a plastic sorting system. Take-back schemes can be used to collect devices distributed in home settings.^130^ Changes in local and regional regulations are needed to allow more circularity in the sector, including the radical possibility to compost or biodigest some point-of-care single diagnostics after sterilisation. These new approaches to hospital waste management require further research and new considerations regarding waste management workflow and training, as well as related policy and legal matters.

## Conclusion

There is a growing awareness of the challenge of diagnostic waste in the global health community, but the proposed solutions focus on the country level, *i.e.*, by improving waste management infrastructure, or enhancing the quality of incinerators. Green procurement schemes focused on products' environmental sustainability (*e.g.*, that of the Global Fund) are on the rise, but if there are no green products available, their impact will be limited. Meanwhile, due to the lack of economic incentives and regulatory hurdles, there has been little appetite among POC providers for investing in developing more sustainable and safer POCT devices.

While this review focuses on the consumable element of POCTs, it is worth noting that the consumable is only one part of the POCT process. Packaging and instrumentation should also be considered as well. However, this review of sustainable POCTs is a first step, helping to highlight the importance of innovation for sustainable and safe diagnostics. The opportunities and challenges associated with mass manufacturing and high-volume deployment of POCTs reveal the intersection of environmental and human health crises, in a world increasingly vulnerable to the impacts of climate change and global pandemics and demonstrate the importance of a planetary health approach to biomedical innovation.

Design for sustainability and safety should be on everyone's mind and researchers, healthcare providers, policy makers and regulators, funders, industrials, charity and civil society all have a duty to create sustainable and safe innovations and a role to play. Importantly, sustainability should not be a ‘siloed’ academic activity, and interdisciplinarity is crucial.

Embedding sustainability and safety considerations at an early stage is key to reduce the impact that future technologies will have on the environment. In addition, it will help to reduce GHG emissions, reduce dependence on fossil resources and support the achievement of a net-zero CO_2_ emission by 2050, which is essential to deliver on post-Paris (COP 21) climate commitments. The rationale behind material choice for the manufacturing of a new microfluidic device has always been driven by physical and chemical properties and prototyping technologies. It should not perhaps come as a surprise that over 20 petrochemical-derived polymers are currently favoured by both academic laboratories and industries in the R&D of microfluidic techniques for point-of-care diagnostics because of their performance, reliability and well controlled manufacturing processes.

The adoption of recycled or bio-derived materials in the field of single-use POCT diagnostics will form strong foundations to pursue the reduction of CO_2_ emissions and plastic pollution in places of use. It is important to underline as well that the material choice cannot be decoupled from consideration of and actions around how the used material is manufactured and disposed of. The potential for circularity in the field of single-use medical consumable is very slowly emerging, but with adequate funding, technological advances and regulatory support could constitute a radical new approach to waste management in the sector.

All actors of the POCT market share responsibility for the waste generated from innovative microfluidic technologies and need to be a part of the solution. Moreover, some sustainable materials appropriate for POCT manufacture already exist and several academic and industry groups have sustainable POCT applications in the pipeline, even if such initiatives have received little visibility.

Although regulatory change is anticipated in the near future, POCT providers will remain reluctant to engage unless they can see tangible technical feasibility and economic benefits, without compromise on diagnostic performance. Therefore, we are in need of models to show that it is technically possible and commercially viable to develop single-use diagnostics using local and sustainable materials, and this initiative should be collaboratively supported by researchers within academia, the point-of-care diagnostic industry and end-users alike.

## Author contributions

This work was conceptualised by A. E. O., A. S. and M. K. K., A. E. O., A. S. and M. K. K. wrote the original draft. Z. N., E. S., C. O. and P. O. reviewed and edited the manuscript.

## Conflicts of interest

There are no conflicts of interest to declare.

## Supplementary Material
